# Graft Versus Host Diseases Presenting Endoscopically as a Tubular Structure Mimicking a Worm

**DOI:** 10.4021/gr2009.10.1320

**Published:** 2009-09-20

**Authors:** Amer A Alkhatib, Maia M Ahdab

**Affiliations:** a Division of Gastroenterology, Department of Medicine, University of Utah, Salt Lake City, Utah, USA

## To the Editor:

Graft Versus Host Diseases (GVHD) is the leading cause of morbidity and mortality after allogenic hematopoietic stem cell transplantation, occurring in up to 75% of patients [1]. According to the degree of involvement in each of the organ systems, acute GVHD can be clinically classified as grades I - IV [2]. Colonoscopic examination can show diffuse edema, hyperemia, patchy erosion, scattered ulcer, sloughing and active bleeding [3, 4]. We report a case of a patient with GVHD that presented endoscopically as a tubular structure that looked like a dead worm.

A 63 years old female patient with history of acute myelogenous leukemia underwent unrelated donor graft rejection followed by matched unrelated donor peripheral blood stem cell transplant presented with diarrhea. The patient underwent colonoscopy that showed mildly congested and erythematous colonic mucosa. Biopsy from the colon showed focal crypt abscess, apoptotic bodies, cryptal destruction and laminar propria fibrosis consistent with graft versus host disease grade 2. CT of the abdomen and pelvis was done and showed a bowel wall thickening due to edema involving multiple loops of jejunum, however the mucosa remained enhancing. The patient was diagnosed based on the clinical presentation with severe gut GVHD.

**Figure 1:**
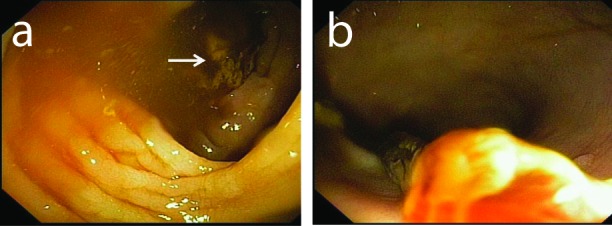
(a) Endoscopic image showing a floating tubular structure inside the colon; (b) Endoscopic image showing the tubular structure is attached to the colonoscopy.

**Figure 2:**
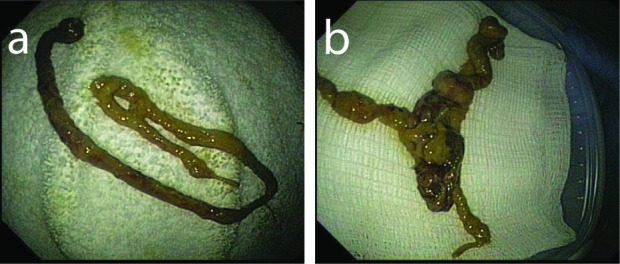
(a, b) Images of the tubular structure that mimic worm on a towel.

Because the patient continued to have diarrhea and work up for infectious diarrhea was negative, a repeated colonoscopy was done and showed a granular mucosa with ulcerations involving the entire the colon. A tubular structure was seen floating in the colon and looked like a worm (Fig. 1). It was 30 cm in length with 0.7 cm in diameter. It was retrieved by the colonoscopy (Fig. 2). A cross sectioning revealed fibrous tissue bands surrounded by clotted and hemorrhagic tissue. The colonic biopsy showed GVHD. The histopathological examination of the tubular structure was consistent with fibrinopurlent exudate.

We concluded that the tubular structure represented a sloughed gut tissue induced by severe gut GVHD.
